# Pseudomonas boreofloridensis sp. nov., and Pseudomonas citrulli sp. nov., isolated from watermelon in Florida

**DOI:** 10.1099/ijsem.0.006596

**Published:** 2024-12-03

**Authors:** Kiersten R. Fullem, Michelle P. MacLellan, Mousami Poudel, Erica M. Goss, Neha Potnis, Gerald V. Minsavage, Jeffrey B. Jones, Mathews L. Paret

**Affiliations:** 1 Department of Plant Pathology, University of Florida, Gainesville, FL, USA; 2 Department of Plant Pathology, University of Georgia, Tifton, GA, USA; 3 Department of Entomology and Plant Pathology, Auburn University, Auburn, AL, USA

**Keywords:** bacteria, cucurbits, *Pseudomonas*, plant pathology, watermelon

## Abstract

Three fluorescent bacterial strains, K1, K13 and K18, were obtained from watermelon (*Citrullus lanatus*) foliage symptomatic of bacterial leaf spot of cucurbits in Florida. The strains underwent phenotypic characterization, including LOPAT (levan production, oxidase activity, pectolytic activity on potato, arginine dihydrolase production and hypersensitive response (HR) on both tobacco and tomato) and pathogenicity testing on watermelon and squash seedlings. Whole-genome sequencing of the isolates was performed, and multi-locus sequence analysis (MLSA) utilizing housekeeping genes *gltA*, *rpoD*, *gapA* and *gyrB* placed the isolates into two distinct clades within the *Pseudomonas* genus. Average nucleotide identity based on blast (ANIb) was used to compare the isolates to *Pseudomonas* reference genomes. Using ANIb, the closest relatives to the novel strains were identified as *Pseudomonas wayambapalatensis* (K1 : 82.58%; K13 : 83.77%) and *Pseudomonas kilonensis* (K18 : 87.16%), although ANIb values were below the 95% threshold for species delineation. DNA–DNA hybridization (genome–genome distance calculation method), comparison to the online Type Genome Server, Biolog biochemical profiling and matrix-assisted laser desorption/ionization time-of-flight mass spectrometry were also unable to identify the isolates as any known species of *Pseudomonas*. Based on the combination of genetic and phenotypic data, we conclude that these isolates represent two novel *Pseudomonas* species, for which we propose the names *Pseudomonas boreofloridensis* sp. nov. (K1, K13^T^, NCPPB 4759=LMG 33364) and *Pseudomonas citrulli* sp. nov. (K18^T^, NCPPB 4761=LMG 33365). The specific epithet *boreofloridensis* was chosen for the geographic location of isolation (northern Florida), while *citrulli* designates the host of origin (*C. lanatus*).

## Introduction

The *Pseudomonas* genus is large and complex and currently contains over 330 validly published species, the most of any Gram-negative bacterial genus [1,2]. Members of *Pseudomonas* are ubiquitous and occupy an incredibly diverse range of hosts and environmental niches, including humans, animals, plants, soil, marine and fresh water and clouds [3,5]. They also display great variation in phenotypic and genomic properties, all of which complicate their classification. The *Pseudomonas* genus has undergone much taxonomic revision since its establishment in 1894, alongside the constant development of new methods of classification [1,5,7]. Prior to the 1960s, prokaryotic taxonomy relied solely on phenotypic characteristics; however, with the advent of DNA-based approaches, DNA–DNA hybridization (DDH) and 16S rRNA gene analysis became standard practices [5,7, 8]. Nonetheless, both methods have their limitations. Previously considered the standard for bacterial classification, traditional DDH analysis has many constraints, including being difficult to perform and the inability to use databases for genome comparisons, and so it is no longer widely used [8,10]. 16S rRNA gene analysis, while useful for rapid preliminary identification of prokaryotic species and required for the description of new bacterial taxa [10,11], has been found to lack the discriminatory power to distinguish between closely related species [1,7, 9, 10]. Today, more precise genetic approaches, such as multi-locus sequence analysis (MLSA) based on housekeeping genes, and whole-genome-based approaches, including average nucleotide identity, and *in silico* DDH (isDDH), serve as valuable tools for bacterial classification [1,5, 7, 9]. Additional phenotypic or physiological analyses, such as Biolog microbial identification test panels, cellular fatty acid profiles and matrix-assisted laser desorption/ionization time-of-flight mass spectrometry (MALDI-TOF MS), are often used in conjunction with the above-described genetic methods of identification for a multiphasic approach [5,12,14].

*Pseudomonas syringae* is a large species complex consisting of 13 phylogroups and more than 60 pathovars [15,16]. Together, its strains exhibit an incredibly diverse host range, including nearly all agriculturally significant crops, and are responsible for many important plant diseases [17,18]. The species is the most commonly reported causal agent of bacterial leaf spot (BLS) of cucurbits, a disease that is present in cucurbit production worldwide and is often observed in the southeastern United States [19,21]. On cucurbits, BLS symptoms vary by species and cultivar but generally consist of tan to brown necrotic foliar lesions, which may appear water-soaked and/or be surrounded by chlorotic halos. Under favorable environmental conditions, lesions expand and coalesce, blighting foliage and potentially leading to delayed fruit maturity, yield losses and death of transplants in the field [20,22, 23].

Between 2020 and 2022, a study was conducted to characterize *Pseudomonas* isolates obtained from cucurbit crops symptomatic for BLS in the southeastern United States [21]. Isolates were collected by sampling field outbreaks as well as symptomatic plants submitted to the University of Florida Plant Diagnostic Clinics. While approximately half of the isolates collected were identified as *P. syringae*, additional *Pseudomonas* species were also documented, and multiple isolates, including three designated K1, K13 and K18, could not be identified as any existing *Pseudomonas* species. Isolates K1 and K13 were obtained from symptomatic watermelon (*Citrullus lanatus*) foliage from a 2020 field outbreak of BLS in northern Florida. Leaves of affected plants exhibited large dark-brown necrotic lesions surrounded by chlorotic halos. Individual lesions were excised from sampled leaves using a sterile razor blade; tissue from lesion margins was then macerated in a small amount of sterile water. The resulting suspension was plated onto nutrient agar (NA, Difco) and incubated for 48 h at 28 °C, after which colonies were transferred with a sterile loop to new plates to obtain pure cultures. Strain K18 was also isolated from symptomatic watermelon foliage collected in 2020 from a different area in northern Florida. The strain was provided by the University of Florida Plant Diagnostic Clinic in Gainesville, FL, where it was isolated.

All three strains were found to fluoresce under UV light when grown on King’s Medium B and were determined to be Gram-negative using a standard potassium hydroxide test [24]. Strains were phenotypically characterized using LOPAT tests, including assays for levan production, oxidase activity, pectolytic activity on potato, arginine dihydrolase production and hypersensitive response (HR) on both tobacco and tomato using needle infiltration of plants [25]. All three strains were found to be oxidase positive, pectolytic activity negative, arginine dihydrolase positive and tobacco HR negative (Table 1). However, K18 was found to produce mucoid growth on 5% sucrose media, indicating levan production, while K1 and K13 were negative for mucoid growth. Additionally, strains K1 and K13 were found to elicit an HR in tomato, while K18 was tomato HR negative. Pathogenicity of strains was assessed through inoculation of watermelon (*C. lanatus*) and squash (*Cucurbita pepo* subsp. *pepo*) seedlings. Bacterial suspensions of 10^8^ c.f.u. ml^−1^ were applied via spray until run-off, after which plants were placed into moistened polyethylene bags and allowed to incubate in a greenhouse (day temperature=28 °C and night temperature=24 °C) for 72 h. After 72 h, no disease symptoms were observed.

**Table 1.** Tests for levan production, oxidase activity, pectolytic activity, arginine dihydrolase production and HR in both tobacco and tomato (LOPAT) for novel strains isolated from watermelon

	*P. boreofloridensis*		*P. citrulli*
Assay	K1	K13^T^	K18^T^
Levan production	−	−	+
Oxidase activity	+	+	+
Pectolytic activity	−	−	−
Arginine dihydrolase activity	+	+	+
Tobacco HR	−	−	−
Tomato HR	+	+	−

DNA was extracted from strains using the Wizard Genomic DNA Purification kit (Promega) and sent to the Microbial Genome Sequencing Center (Pittsburgh, PA, USA) for whole-genome sequencing using the Illumina NextSeq 2000 platform. Resulting genetic reads were assembled with a pipeline utilizing genome assembly tool SPAdes (v. 3.10.1) [26]. 16S rRNA gene sequences were used for preliminary identification of strains and were compared using blastn to both National Center for Biotechnology Information (NCBI) databases, and a database comprising 16S rRNA sequences from the type strains of all *Pseudomonas* species validly published at the time of analysis for which genetic data could be found (LPSN, *n*=329) [2]. As a result of this analysis, the highest percent identities for the 16S rRNA gene sequence were found between K1 and K13 and *P. parafulva* (strain NBRC 16636^T^, accession number GCF_000730645.1, both 99.60%) and K18 and *P. citri* (strain OPS13-3 ^T^, accession number GCF_025336485.1, 99.40%). The results of 16S rRNA sequence analysis are located in Table 2.

**Table 2.** Genomic relationships, including 16S rRNA gene sequence similarity, average nucleotide identity based on blast (ANIb) and DDH isDDH between type strains of novel species (*P. boreofloridensis* K13^T^ and *P. citrulli* K18^T^) and those of closely related *Pseudomonas* species [2]

*P. boreofloridensis* K13^T^			
**Species**	16S rRNA gene sequence similarity (%)	ANIb (%)	isDDH (%)
*P. boreofloridensis* K1	100	99.99	100
*P. wayambapalatensis* RW3S1^T^	98.91	83.77	29.9
*P. muyukensis* COW39^T^	98.96	82.53	41.8
*P. peradeniyensis* BW13M1^T^	98.70	82.51	28
*P. xantholysinigenes* RW9S1A^T^	98.83	82.38	27.6
*P. sichuanensis* WCHPs060039^T^	98.89	82.18	36.3
*P. maumuensis* COW77^T^	98.96	82.12	42.8
*P. anuradhapurensis* RD8MR3^T^	98.54	81.96	27.5
*P. fakonensis* COW40^T^	98.83	81.96	27.7
*P. asiatica* RYU5^T^	99.02	81.94	33.3
*P. plecoglossicida* FPC-951^T^	98.86	81.92	36.2
*P. inefficax* JV551A3^T^	98.96	81.53	32.6
*P. vlassakiae* RW4S2^T^	98.87	81.4	26.6
*P. kurunegalensis* RW1 P2^T^	99.05	81.31	26.3
*P. juntendi* BML3^T^	99.09	81.29	33.2
*P. urmiensis* SWRI10^T^	99.22	81.08	36.4
*P. kermanshahensis* SWRI100-2^T^	99.02	81.05	32.9
*P. promysalinigenes* RW10S1^T^	99.02	80.55	25
*P. parafulva* NBRC 16636^T^	99.60	80.49	37.8
*P. shirazensis* SWRI56-8^T^	98.96	80.08	33.4
*P. fulva* IAM 1529^T^	98.95	79.9	33.3
*P. laurentiana* GSL-010^T^	99.05	77.67	24.1
***P. citrulli* K18^T^**			
**Species**	**16S rRNA gene sequence similarity (%**)	**ANIb (%**)	**isDDH (%**)
*P. kilonensis* 520-20^T^	98.53	87.16	35.3
*P. beijingensis* FP830^T^	98.76	87.01	35
*P. ogarae* strain SWRI108^T^	98.63	86.93	35.4
*P. brassicacearum* DBK11^T^	98.24	86.93	35.6
*P. bijieensis* L22-9^T^	98.76	86.81	34.7
*P. thivervalensis* SBK 26^T^	98.6	86.76	34.9
*P. zanjanensis* SWRI12^T^	98.83	86.48	34.4
*P. viciae* 11K1^T^	98.77	86.35	34.7
*P. mediterranea* CFBP 5447^T^	98.06	86.08	33.3
*P. citri* OPS13-3^T^	99.40	85.95	33.7
*P. alvandae* SWRI17^T^	98.89	85.58	32.2
*P. marvdashtae* SWRI102^T^	98.76	85.57	32.1
*P. koreensis* Ps 9-14^T^	98.35	81.62	26.6
*P. gregormendelii CCM* 8506^T^	98.24	81.36	26.5
*P. granadensis* F-278^T^	99.22	81.12	25.7
*P. shahriarae* SWRI52^T^	98.89	80.33	25.3
*P. gessardii* CIP-105469^T^	98.81	80.24	25.4
*P. proteolytica* CMS 64^T^	98.28	80.21	25.3
*P. azadiae* strain SWRI103^T^	98.76	80.12	24.9
*P. pergaminensis* 1008^T^	98.76	79.86	25
*P. khavaziana* SWRI124^T^	98.76	79.78	25

Pairwise average nucleotide identity based on blast (ANIb) and isDDH were performed using the genomes of the three novel strains and the strains to which they shared the highest percent similarities of the 16S rRNA gene sequence (Table 2). ANIb and isDDH were calculated using the web tools JSpecies (v. 4.1.1, http://jspecies.ribohost.com/jspeciesws) and genome–genome distance calculator (formula 2, v. 3.0, http://ggdc.dsmz.de/home.php), respectively [27,28]. Previous research has established an ANI value of 95% or above as the threshold for prokaryotic species delineation and equivalent to the recommended threshold of 70% or greater for DDH [29]. Strains K1 and K13 were determined to share 99.99% ANIb, and so belong to the same species. The highest ANIb values when compared to reference strains were found between K1 and K13 and *P. wayambapalatensis* (strain RW3S1^T^, accession number CP077096.1, 82.53%; 83.77%) and K18 and *P. kilonensis* (strain 520-20^T^, accession number GCF_001269885, 87.16%) (Table 2). These values are considerably lower than the 95% threshold value used for species identification and suggest that the novel strains may belong to two currently undescribed species of *Pseudomonas.* isDDH results supported those of ANIb, with the highest similarities found between strains K1 and K13 to *P. maumuensis* (strain COW77^T^, accession number CP077077.1, both 42.80%) and K18 to * P. brassicacearum* (strain DBK11^T^, accession number GCF_012034345, 35.60%), all well below the 70% DDH threshold for species identification (Table 2).

**Fig. 1.**
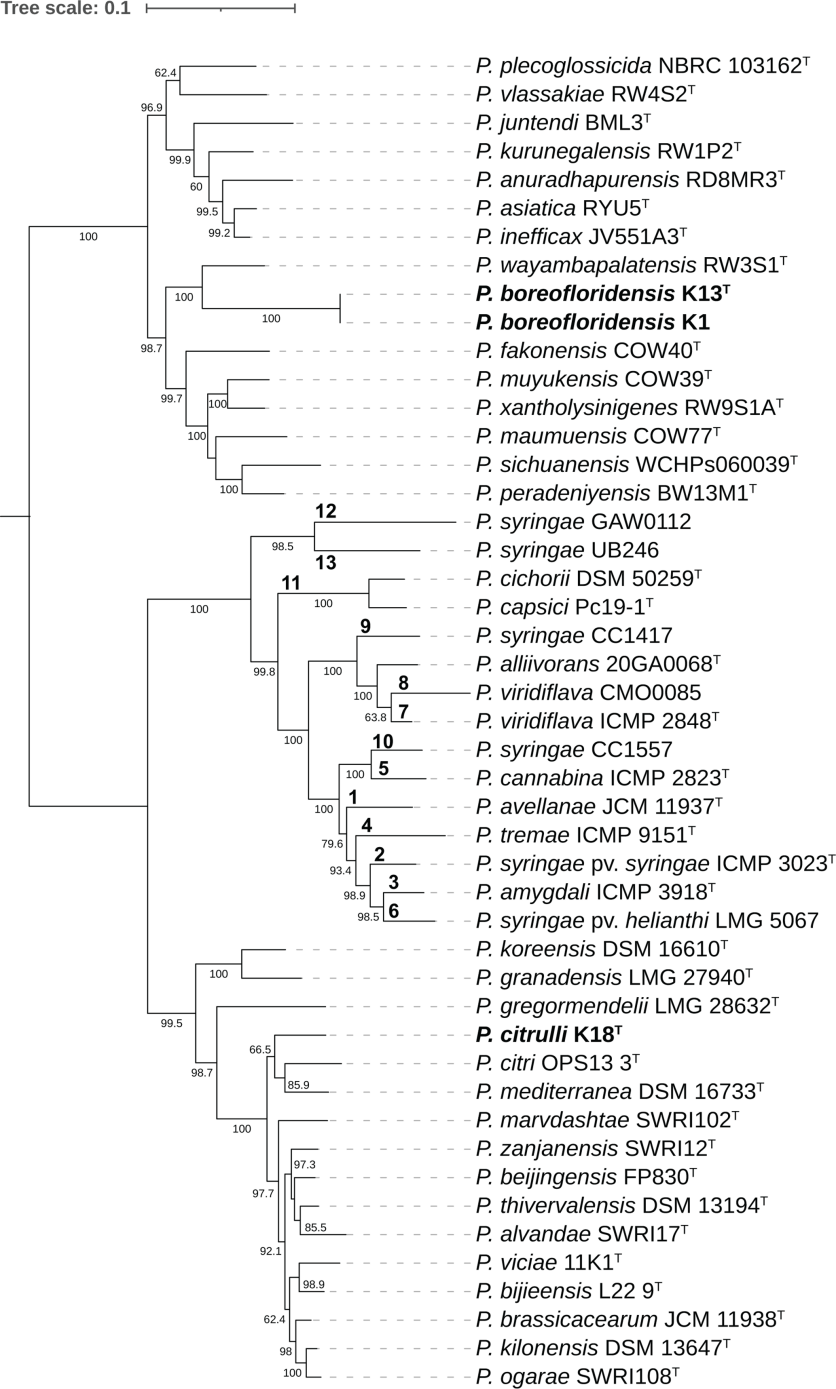
Maximum-likelihood phylogenetic tree based on concatenated alignments of housekeeping genes *gltA*, *rpoD*, *gapA* and *gyrB.* Figure 1 Footnote: Bootstrap values based on 100 replicates are indicated at branching points. Bolded numbers designate *P. syringae* species complex (Pssc) phylogroups.

The *P. syringae* species complex (Pssc) contains both the most common causal agent of BLS, *P. syringae*, and multiple additional *Pseudomonas* species associated with the disease [21] and has been differentiated into 13 phylogroups based on MLSA of housekeeping genes *cts* (*gltA*), *rpoD*, *gapA* and *gyrB* [16]. To determine the phylogenetic placement of the novel strains within the *Pseudomonas* genus, and in relation to the Pssc, MLSA utilizing the aforementioned gene sequences was performed comparing novel strains to strains representing the Pssc phylogroups as well as to *Pseudomonas* species to which they were found to share the highest percent ANIb. Analysis was performed using the program AutoMLSA2 (v. 0.8.1) with *Pseudomonas* reference genomes obtained from the NCBI or gene sequences obtained from the Plant Associated and Environmental Microbes Database (PAMDB) [30]. A maximum-likelihood tree (Fig. 1) was constructed using IQ-TREE (v. 2.1.3) [31] and visualized using iTOL (v. 6.9) [32] and Adobe Illustrator. Phylogenetic analysis placed the three novel strains into two separate clades within the genus *Pseudomonas*, but outside of the *P. syringae* species complex. K1 and K13 were placed into the same clade, which was closest to, though distinct from, that of *P. wayambapalatensis* (strain RW3S1^T^). Strain K18 was placed in its own clade, which was sister to another containing *P. citri* (strain OPS13-3^T^) and *P. mediterranea* (strain DSM 16733^T^, accession number GCF_900106005.1).

To provide additional support for the identification of the strains as novel species, genomes of strains K13 and K18 were compared to the online Type Genome Server (TYGS, https://tygs.dsmz.de/), a high-throughput web tool for prokaryotic genome-based taxonomy, which utilizes isDDH and the Genome Blast Distance Phylogeny approach to determine genomic and phylogenetic relationships between user-uploaded genomes and those of prokaryotic type strains [33]. TYGS was unable to identify isolates K13 and K18 as members of any species contained within its database. Whole-genome phylogenies based on isDDH comparisons and 16S rRNA gene sequence phylogenies generated by the program containing the novel strains and their closest identified relatives are included within Figs S1–S4.

**Fig. 2.**
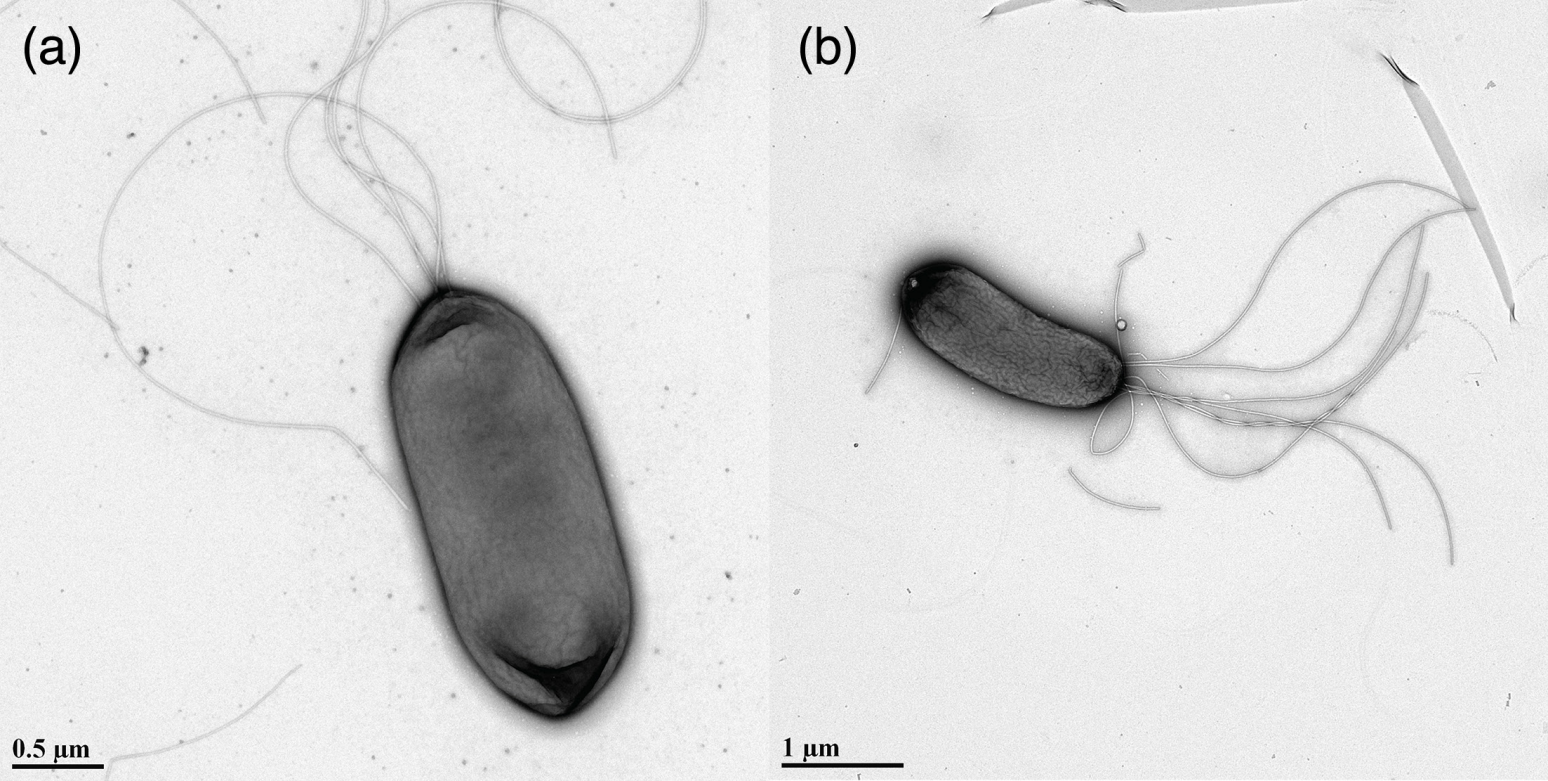
Images of cells of (**a**) *P. boreofloridensis* K13^T^ and (**b**) *Pseudomonas citrulli* K18^T^ as captured by a Tecnai G2 Spirit TWIN 120 kV transmission electron microscope, University of Florida’s Interdisciplinary Center for Biotechnology Research (ICBR).

Based on the sum of the results of the aforementioned analyses, we propose the creation of two new species within the genus *Pseudomonas*, designated as *Pseudomonas boreofloridensis* (K1, K13^T^) and *Pseudomonas citrulli* (K18^T^). Further phenotypic characterization was conducted to describe the type strains of each proposed species and provide additional support for their identities as novel species. Bacterial cultures were grown overnight in nutrient broth (Difco) before undergoing imaging at the University of Florida’s Interdisciplinary Center for Biotechnology Research (ICBR), using a Tecnai G2 Spirit TWIN 120 kV transmission electron microscope (Fig. 2). Cells of both proposed species were determined to be rod-shaped and to possess lophotrichous flagella.

Strains were also phenotypically profiled using the Biolog Gen III MicroPlate system (S5). Bacterial cultures of each strain were grown overnight on Biolog Universal Growth agar (Biolog Inc.); a small amount of bacteria was then suspended in Biolog inoculation fluid (Biolog Inc.) to achieve the level of turbidity recommended by the Biolog protocol. For each strain, 100 µl of bacterial suspension was added per well to a MicroPlate; plates were then incubated at 28 °C for 24 h, after which results were recorded and compared to the Biolog database. The type strains of both proposed species were identified by the program as members of the genus *Pseudomonas*, although they were unable to be further identified as members of any existing species contained within the database.

Bacterial cultures were sent to Charles River Laboratories (Newark, DE, USA) for MALDI-TOF MS. Resulting spectra were compared to Bruker Biotyper (v. 11758) and Charles River spectral reference libraries (v. 23.01) for species identification. Charles River provides probable species identification for a sample if the similarity score between the unknown isolate and a reference strain is greater than or equal to 1.75. Spectra produced from *P. boreofloridensis* type strain K13 did not meet this requirement, and so, the isolate was unable to be identified as any existing bacterial species. *P. citrulli* sp. nov. type strain K18 was identified as a member of the *Pseudomonas fluorescens* group, which is described by Charles River Laboratories as a group of very closely related species that cannot be reliably differentiated by MALDI-TOF MS. *Pseudomonas chlororaphis* (similarity score 2.110) and *P. tolaasii* (similarity score 1.930) were proposed as the most likely identities of the strain; however, ANIb analysis comparing the type strains of these species to that of * P. citrulli* produced values below the 95% threshold for species identification (*P. chlororaphis*: 82.34%, *P. tolaasii*: 80.07%).

In summary, multiphasic analyses, including phylogeny based on multi-locus sequence of housekeeping genes, calculation of ANIb and isDDH values based on whole-genome sequences, comparison of genomes to the online TYGS, biochemical profiling with the Biolog Gen III MicroPlate system and analysis with MALDI-TOF MS, show that bacterial strains isolated from Florida watermelon foliage represent two novel species in the genus *Pseudomonas*. For these, we propose the names *P. boreofloridensis* and *Pseudomonas citrulli*.

## Description of *Pseudomonas boreofloridensis* sp. nov.

*Pseudomonas boreofloridensis* (bor.e.o.flor.id.en’sis. Gr. masc. n. *boreas*, the north; N.L. fem. adj. *floridensis*, of or from Florida; N.L. fem. adj. *boreofloridensis*, pertaining to northern Florida, the geographical location of isolation).

Cells are Gram-negative motile rods (2.0–2.40 µm in length and ~1.0 µm in width) with lophotrichous flagella. Colonies are smooth, circular, light yellow in color and 2.0–2.5 mm in diameter when grown on NA and incubated for 48 h at 28 °C. Colonies produce a diffusible fluorescent pigment when grown on King’s medium B. Strains are negative for levan production and pectolytic activity and do not elicit an HR in tobacco. They are positive for oxidase and arginine dihydrolase activities and elicit an HR in tomato. Inoculation of watermelon (*C. lanatus*) and squash (*C. pepo* subsp. *pepo*) seedlings with * P. boreofloridensis* strains did not lead to disease development. Strain K13^T^ was observed to grow at pH 5 and 6, as well as at salinity levels of 1 and 4% NaCl, though not at 8% NaCl. Growth of isolates suspended in tryptic soy broth (Sigma-Aldrich) was assessed at incubation temperatures of 0, 4, 15, 21, 28, 32, 37 and 41 °C. After 48 h of incubation, growth was observed only in cultures incubated at temperatures between 15 and 37 °C, with optimal growth occurring between 21 and 32 °C. The type strain, K13^T^ (NCPPB 4759^T^ = LMG 33364^T^), was isolated from watermelon foliage symptomatic for BLS of cucurbits in Florida. The NCBI GenBank accession numbers for the genome assemblies of *Pseudomonas boreofloridensis* sp. nov. strains are: K1, GCF_041947115.1 and K13^T^, GCF_030580795.1. The accession numbers for 16S rRNA gene sequences are K1, OR725067 and K13^T^, OR725079.1. The G+C content of strains K13^T^ and K1 was found to be 63.49%; genome statistics for all strains are provided in Table 3.

**Table 3.** Genome statistics, including genome coverage, genome length, N50 value, G+C content and number of contigs for *P. boreofloridensis* and *P. citrulli* strains

	*P. boreofloridensis*		*P. citrulli*
	**K1**	**K13^T^**	**K18^T^**
Genome coverage	76	93	69
Genome length (Mb)	5.13	5.13	5.83
N50 value	289,564	343,525	108,455
G+C content (mol%)	63.49	63.49	62.83
Contigs	56	49	100

## Description of *Pseudomonas citrulli* sp. nov.

*Pseudomonas citrulli* (ci.trul’li. N.L. gen. n. *citrulli*, pertaining to the host of origin, *Citrullus lanatus*).

Cells are Gram-negative motile rods (1.75–2.25 µm in length and ~0.75 µm in width) with lophotrichous flagella. Colonies are smooth, circular, white-cream in color and 1.0–1.5 mm in diameter when grown on NA and incubated for 48 h at 28 °C. Colonies produce a diffusible fluorescent pigment when grown on King’s medium B. The type strain is negative for pectolytic activity and does not elicit a hypersensitive reaction in tobacco or tomato. It is positive for levan production, oxidase activity and arginine dihydrolase activity. Inoculation of watermelon (*C. lanatus*) and squash (*C. pepo* subsp. *pepo*) seedlings with *P. citrulli* did not lead to disease development. Strain K18^T^ was found to grow at pH 6, though not at pH 5. Additionally, the strain grew when exposed to salinity levels of 1 and 4% NaCl, though not at 8% NaCl. Growth of isolates suspended in tryptic soy broth (Sigma-Aldrich) was assessed at incubation temperatures of 0, 4, 15, 21, 28, 32, 37 and 41 °C. After 48 h of incubation, growth was observed only in cultures incubated at temperatures between 15 and 37 °C, with optimal growth occurring between 21 and 32 °C. The type strain, K18^T^ (NCPPB 4761^T^ = LMG 33365^T^), was isolated from watermelon foliage symptomatic for BLS of cucurbits in Florida. The NCBI GenBank accession number for the genome assembly of *Pseudomonas citrulli* sp. nov. K18^T^ is GCF_030580855.1. The accession number for the 16S rRNA gene sequence of strain K18^T^ is OR725083. The G-C content of strain K18^T^ was found to be 62.83%; genome statistics for all strains are provided in Table 3.

## Supplementary material

10.1099/ijsem.0.006596 Uncited Supplementary Material 1.

## Abbreviations

ANIb: average nucleotide identity based on blast
BLS: bacterial leaf spot
DDH: DNA–DNA hybridization
HR: hypersensitive response
isDDH: in silico DNA–DNA hybridization
MLSA: multi-locus sequence analysis
TYGS: type genome server
