# Corrigendum: Pseudomonas boreofloridensis sp. nov., and Pseudomonas citrulli sp. nov., isolated from watermelon in Florida

**DOI:** 10.1099/ijsem.0.007147

**Published:** 2026-04-21

**Authors:** Kiersten R. Fullem, Michelle P. MacLellan, Mousami Poudel, Erica M. Goss, Neha Potnis, Gerald V. Minsavage, Jeffrey B. Jones, Mathews L. Paret

**Affiliations:** 1 Department of Plant Pathology, University of Florida, Gainesville, FL, USA; 2 Department of Plant Pathology, University of Georgia, Tifton, GA, USA; 3 Department of Entomology and Plant Pathology, Auburn University, Auburn, AL, USA

 After the publication of the previous article [1], it was brought to our attention that the name *Pseudomonas citrulli* is illegitimate as it contravenes Rule 12b of the International Code of Nomenclature of Prokaryotes [2], due to the prior use of the epithet *citrulli* in *Pseudomonas pseudoalcaligenes* subsp. *citrulli* [3,4], now *Acidovorax citrulli* (Schaad *et al.* 1978) Schaad *et al*. 2009 [5,6].

We propose *Pseudomonas citrullilanati* as a replacement name (*nomen novum*) for the illegitimate name *Pseudomonas citrulli*. The species description is as follows:

## *Pseudomonas citrullilanati* Nom. Nov.

*Pseudomonas citrullilanati* (ci.trul.li.la.na’ti. N.L. gen. n. *citrullilanati*, pertaining to the host of origin, *Citrullus lanatus*).

Illegitimate basonym: *Pseudomonas citrulli* Fullem *et al*. 2024.

Cells are Gram-negative motile rods (1.75–2.25 µm in length and ~0.75 µm in width) with lophotrichous flagella. Colonies are smooth, circular, white-cream in colour and 1.0–1.5 mm in diameter when grown on NA and incubated for 48 h at 28 °C. Colonies produce a diffusible fluorescent pigment when grown on King’s medium B. The type strain is negative for pectolytic activity and does not elicit a hypersensitive reaction in tobacco or tomato. It is positive for levan production, oxidase activity and arginine dihydrolase activity. Inoculation of watermelon (*Citrullus lanatus*) and squash (*Cucurbita pepo* subsp. *pepo*) seedlings with strain K18^T^ does not result in disease development. Strain K18^T^ can grow at pH 6, but not at pH 5. Additionally, the strain grew in the presence of 1 and 4% NaCl, but not at 8% NaCl. Growth was observed in TSB in cultures incubated at temperatures between 15 and 37 °C, with optimal growth occurring between 21 and 32 °C.

The type strain, K18^T^ (NCPPB 4761^T^ = LMG 33365^T^), was isolated from watermelon foliage symptomatic for bacterial leaf spot of cucurbits in Florida. The NCBI GenBank accession number for the genome assembly of strain K18^T^ is GCF_030580855.1. The accession number for the 16S rRNA gene sequence of strain K18^T^ is OR725083. The G-C content of strain K18^T^ was found to be 62.83%.

## Abbreviations

TSB: Tryptic soy broth
