# Supplementary material for: Pseudomonas boreofloridensis sp. nov., and Pseudomonas citrulli sp. nov., isolated from watermelon in Florida
Source: Int J Syst Evol Microbiol. 2024 Dec 3;74(12):006596. doi: 10.1099/ijsem.0.006596 (PMC12453567; doi:10.1099/ijsem.0.006596)
Supplement: Uncited Supplementary Material 1. [file ijsem-74-06596-s001.pdf]

S1. A 16S rRNA gene sequence phylogeny including *P. boreofloridensis* K13^T and relevant *Pseudomonas* type strain genomes as produced by the online Type Genome Server (TYGS)

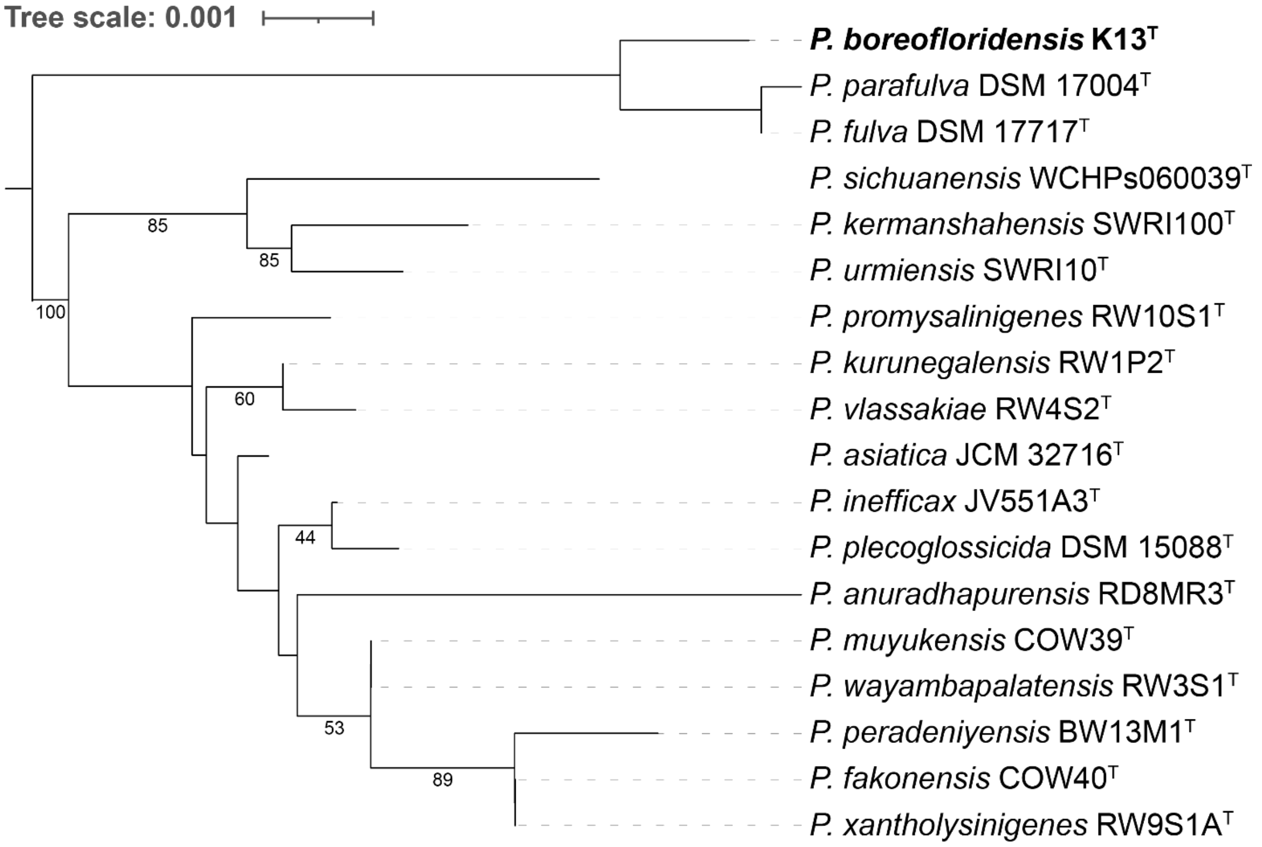

Phylogeny generated using distance formula 5 and distance algorithm CharacterCoverage.

Bootstrap values based on 100 replicates are indicated at branching points.

S2. A whole-genome phylogeny based on isDDH comparisons of *P. boreofloridensis* K13^T and relevant *Pseudomonas* type strain genomes as produced by the online Type Genome Server (TYGS)

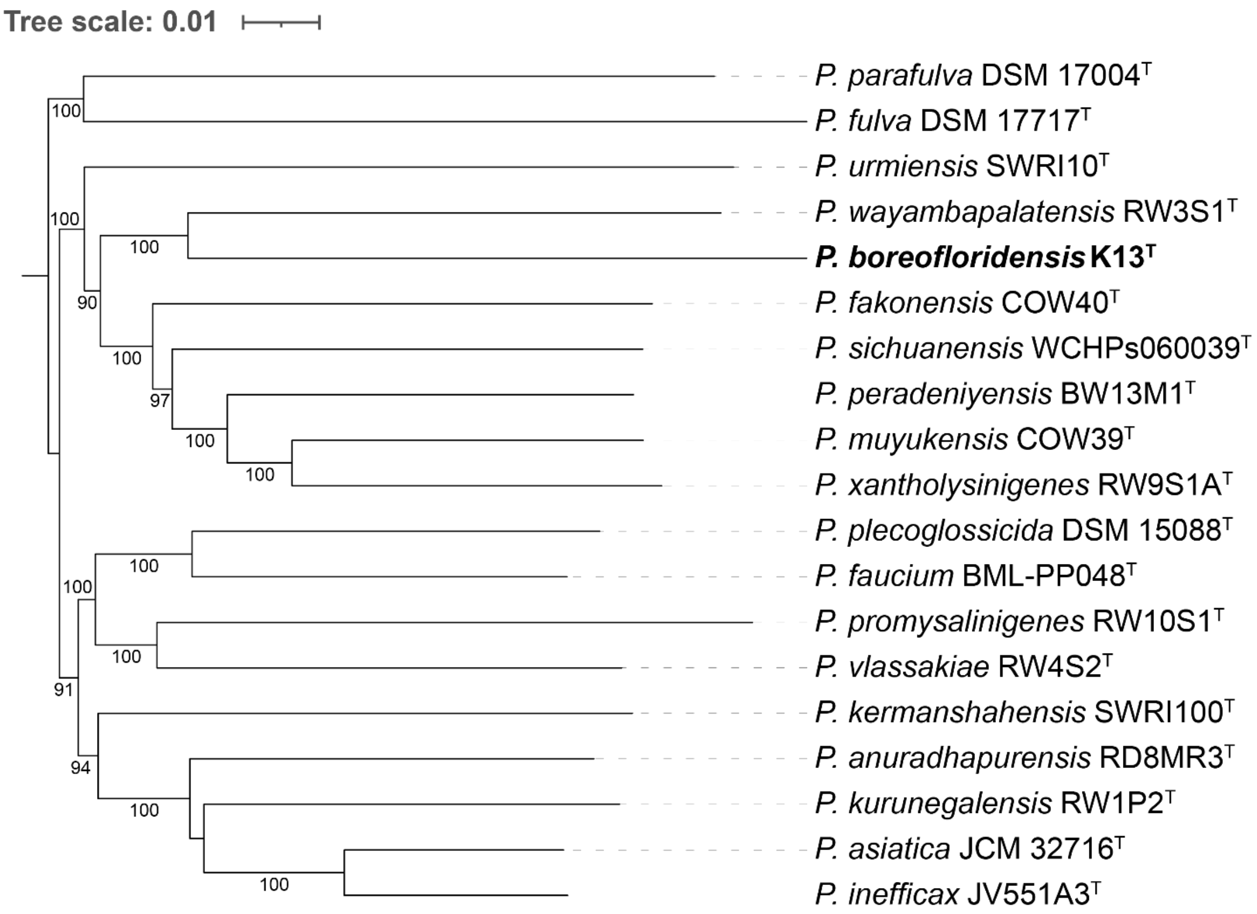

Phylogeny generated using distance formula 5 and distance algorithm GreedyWithTrimming.

Bootstrap values based on 100 replicates are indicated at branching points.

S3. A 16S rRNA gene sequence phylogeny including *P. citrulli* K18^T and relevant *Pseudomonas* type strain genomes as produced by the online Type Genome Server (TYGS)

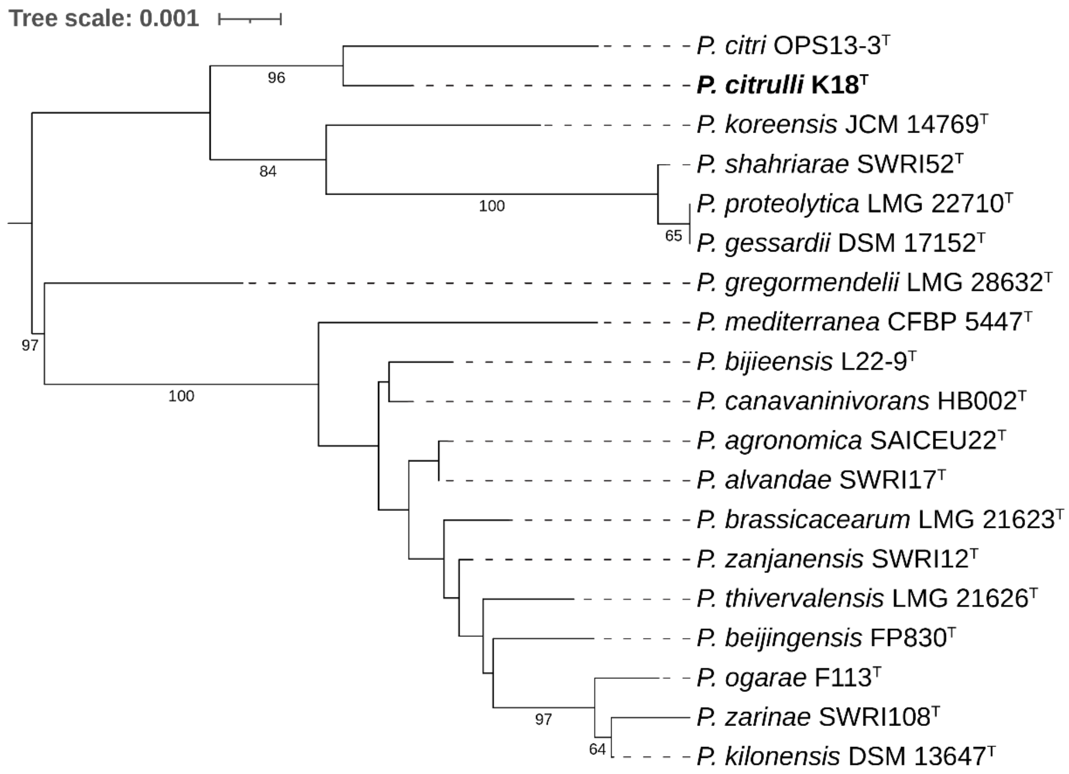

Phylogeny generated using distance formula 5 and distance algorithm CharacterCoverage.

Bootstrap values based on 100 replicates are indicated at branching points.

S4. A whole-genome phylogeny based on isDDH comparisons of *P. citrulli* K18^T and relevant *Pseudomonas* type strain genomes as produced by the online Type Genome Server (TYGS)

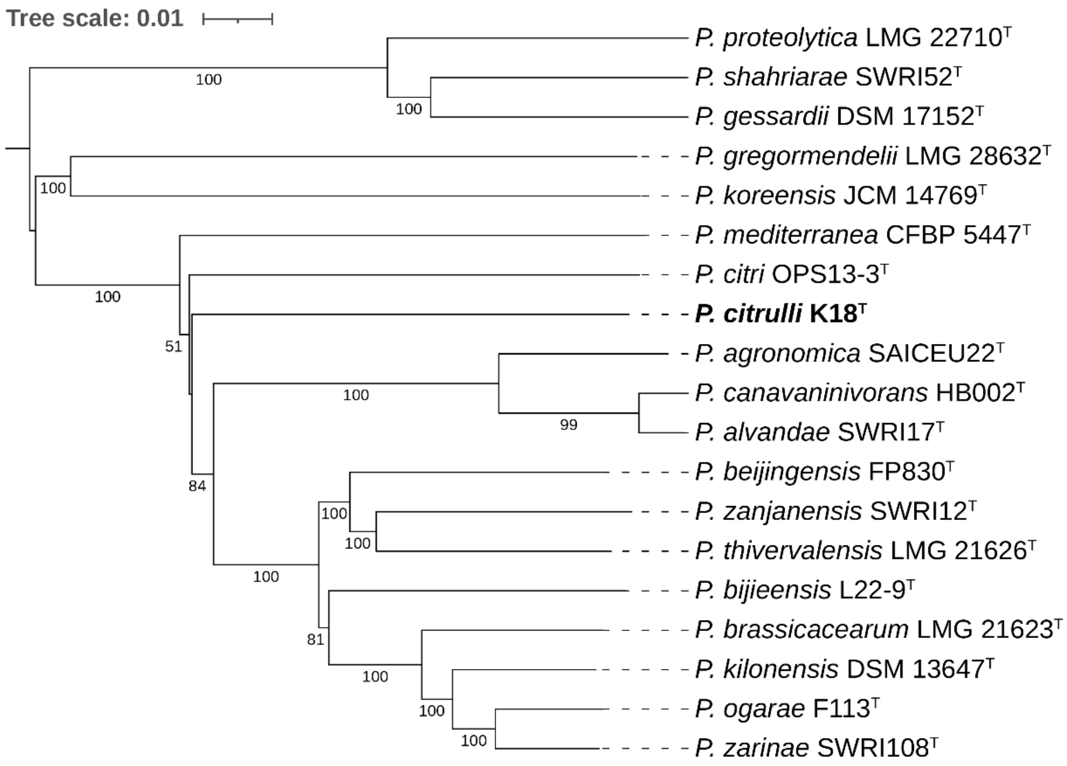

Phylogeny generated using distance formula 5 and distance algorithm GreedyWithTrimming.

Bootstrap values based on 100 replicates are indicated at branching points.

S5. Biolog Gen III assay results for *P. boreofloridensis* and *P. citrulli* type strains

**Carbon Source Utilization:**	***P. boreofloridensis* K13^T**	***P. citrulli* K18^T**
Dextrin	-	-
D-Maltose	-	-
D-Trehalose	-	-
D-Cellobiose	-	-
Gentiobiose	-	+
Sucrose	-	-
D-Turanose	-	+/-
Stachyose	-	-
D-Raffinose	-	-
α-D-Lactose	-	-
D-Melibiose	-	+
β-Methyl-D-Glucoside	-	-
D-Salicin	-	-
N-Acetyl-D-Glucosamine	-	-
N-Acetyl-β-D-Mannosamine	-	-
N-Acetyl-D-Galactosamine	-	-
N-Acetyl Neuraminic Acid	-	-
α-D-Glucose	+	+
D-Mannose	+	+
D-Fructose	-	+
D-Galactose	+/-	+
3-Methyl Glucose	-	+/-
D-Fucose	+	+
L-Fucose	+	+
L-Rhamnose	-	+
Inosine	-	-
D-Sorbitol	-	-
D-Mannitol	-	-
D-Arabitol	-	-
Myo-Inositol	-	-
Glycerol	-	-
D-Glucose-6-PO4	+	-
D-Fructose-6-PO4	-	+
D-Aspartic Acid	-	-
D-Serine	-	-
Gelatin	-	-
Glycyl-L-Proline	-	-
L-Alanine	-	-
L-Arginine	-	-
L-Aspartic Acid	-	-
L-Glutamic Acid	+	-

S5. Continued

**Carbon Source Utilization:**	***P. boreofloridensis* K13ᵀ**	***P. citrulli* K18ᵀ**
L-Histidine	+	-
L-Pyroglutamic Acid	-	-
L-Serine	-	-
Pectin	-	-
D-Galacturonic Acid	-	+
L-Galactonic Acid Lactone	-	+
D-Gluconic Acid	-	-
D-Glucuronic Acid	+	+
Glucuronamide	+	+
Mucic Acid	+	-
Quinic Acid	+	+
D-Saccharic Acid	-	-
p-Hydroxy-Phenylacetic Acid	-	-
Methyl Pyruvate	-	-
D-Lactic Acid Methyl Ester	-	-
L-Lactic Acid	-	-
Citric Acid	+	+/-
α-Keto-Glutaric Acid	+/-	+/-
D-Malic Acid	-	-
L-Malic Acid	+	-
Bromo-Succinic Acid	-	-
Tween 40	-	-
γ-Amino-Butyric Acid	-	-
α-Hydroxy-Butyric Acid	+	-
β-Hydroxy-D,L-Butyric Acid	-	-
α-Keto-Butyric Acid	-	-
Acetoacetic Acid	-	-
Propionic Acid	+	+
Acetic Acid	+	+
Formic Acid	-	-
**Chemical Sensitivity Assays:**		
pH 6	+	+
pH 5	+	-
1% NaCl	+	+
4% NaCl	+	+
8% NaCl	-	-
1% Sodium Lactate	+	+
Fusidic Acid	+	+
D-Serine	+	+
Troleandomycin	+	+
Rifamycin SV	+	+
Minocycline	+	+

S5. Continued

**Chemical Sensitivity Assays:**	***P. boreofloridensis* K13^T**	***P. citrulli* K18^T**
Lincomycin	+	+
Guanadine HCl	+	+
Niaproof 4	+	+
Vancomycin	+	+
Tetrazolium Violet	+	+
Tetrazolium Blue	+	+
Nalidixic Acid	+	+
Lithium Chloride	-	+/-
Potassium Tellurite	+	+
Aztreonam	+	+
Sodium Butyrate	-	+
Sodium Bromate	+	-

Positive reactions are designated (+); negative reactions are designated (-); indeterminate reactions are designated (+/-)
